# Corrigendum to ‘Pre-emptive TIPS should be considered in high-risk patients with both acute variceal bleeding and severe alcohol-related hepatitis^’^ (JHEP Reports 7 [2025] 101611)

**DOI:** 10.1016/j.jhepr.2026.101750

**Published:** 2026-02-11

**Authors:** Marika Rudler, Virginia Hernández-Gea, Hélène Larrue, Charlotte Bouzbib, Bogdan Procopet, Anna Baiges, Fanny Turon, Candido Villanueva, Agustin Albillos, Edilmar Alvarado Tapias, Lise Lott Gluud, Michael Praktiknjo, Joan Genesca, Meritxell Ventura-Cots, Ares Villagrasa, Susanna Rodrigues, Sarah Mouri, Álvaro Giráldez-Gallego, Helena Masnou Ridaura, Wim Laleman, Christophe Bureau, Marie-Angèle Robic, Lukas Hartl, Luis Tellez, Alexander Zipprich, Nuria Canete, Philippe Sultanik, Olivier Deckmyn, Mattias Mandorfer, Marco Senzolo, Filippo Schepis, Dhiraj Tripathi, Juan Carlos Garcia Pagan, Dominique Thabut, a study by the Baveno Cooperation

**Affiliations:** 1 AP-HP, Sorbonne Université, Liver Intensive Care Unit, Hepatogastroenterology Department, La Pitié-Salpêtrière Hospital, Paris, France; 2 INSERM UMR_S 938, Centre de recherche Saint-Antoine, Maladies métaboliques, biliaires et fibro-inflammatoire du foie, Institute of Cardiometabolism and Nutrition (ICAN), Paris, France; 3 Brain-Liver Pitié-Salpêtrière Study Group (BLIPS), Paris, France; 4 Liver Unit, Hospital Clínic, Institut de Investigacions Biomèdiques August Pi i Sunyer (IDIBAPS), Barcelona, Spain; 5 Service d’Hépatologie Hôpital Rangueil CHU Toulouse et Université de Toulouse, Toulouse, France; 6 Department of Gastroenterology, Hospital de Sant Pau and CIBERehd, Barcelona, Spain; 7 Department of Gastroenterology and Hepatology, Hospital Universitario Ramón y Cajal, Instituto Ramón y Cajal de Investigación Sanitaria (IRYCIS), Universidad de Alcalá, Madrid, Spain; 8 Department of Gastroenterology, Hospital Santa Creu i Sant Pau, Autonomous University of Barcelona, Department of Medicine, Biomedical Research Institute Sant Pau (IIB Sant Pau), Barcelona, Spain; 9 Gastro Unit, Copenhagen University Hospital, Hvidovre, Denmark; 10 Department of Medicine I, University Hospital Bonn, Bonn, Germany; 11 Liver Unit, Digestive Diseases Area, Vall d'Hebron University Hospital, Vall d'Hebron Institute of Research (VHIR), Vall d'Hebron Barcelona Hospital Campus, Autonomous University of Barcelona, Barcelona, Spain; 12 Gastroenterology and Hepatology Department, Centro Hospitalar São João, Porto, Portugal; 13 UCM Digestive Diseases and CIBEREHD, University Hospital Virgen del Rocío, Institute of Biomedicine of Seville (CSIC/HUVR/US), University of Seville, Seville, Spain; 14 Division of Gastroenterology and Hepatology, IRCCS Ca' Granda Maggiore Hospital Foundation, University of Milan, Milan, Italy; 15 Department of Gastroenterology and Hepatology, Division of Liver & Biliopancreatic Disorders and Liver Transplantation, University Hospitals Leuven, KU Leuven, Leuven, Belgium; 16 Department of Medicine III, Division of Gastroenterology and Hepatology, Medical University of Vienna, Vienna, Austria; 17 First Department of Internal Medicine, Martin Luther University Halle-Wittenberg, Halle (Saale), Germany; 18 Liver Section, Gastroenterology Department, Hospital del Mar, Universitat Autònoma de Barcelona, Barcelona, Spain; 19 BioPredictive, Paris, France; 20 Unit of Vascular Liver Diseases and Treatment of Portal Hypertension, Gastroenterology, Department of Surgery, Oncology and Gastroenterology, University Hospital of Padua, Padua, Italy; 21 Severe Liver Diseases Departmental Unit (M.E.C.), AOU Policlinico of Modena, University of Modena and Reggio Emilia, Modena, Italy; 22 Liver Unit, University Hospitals Birmingham NHS Foundation Trust, Birmingham Health Partners, Birmingham, UK

In the published version of this article, the name of the author Dr. Virginia Hernández-Gea was incorrectly presented without the hyphen. The correct author name is Virginia Hernández-Gea. The authors apologise for any inconvenience caused.

