# Supplementary material for: Pre-emptive TIPS should be considered in high-risk patients with both acute variceal bleeding and severe alcohol-related hepatitis
Source: JHEP Rep. 2025 Sep 29;7(12):101611. doi: 10.1016/j.jhepr.2025.101611 (PMC12682117; doi:10.1016/j.jhepr.2025.101611)
Supplement: Multimedia component 1 [file mmc1.pdf]

Pre-emptive TIPS should be considered in high-risk patients with both acute variceal bleeding and severe alcohol-related hepatitis☆

Marika Rudler, Virginia Hernandez Gea, Hélène Larrue, Charlotte Bouzbib, Bogdan Procopet, Anna Baiges, Fanny Turon, Candido Villanueva, Agustin Albillos, Edilmar Alvarado Tapias, Lise Lott Gluud, Michael Praktiknjo, Joan Genesca, Meritxell Ventura-Cots, Ares Villagrasa, Susanna Rodrigues, Sarah Mouri, Álvaro Giráldez-Gallego, Helena Masnou Ridaura, Wim Laleman, Christophe Bureau, Marie-Angèle Robic, Lukas Hartl, Luis Tellez, Alexander Zipprich, Nuria Canete, Philippe Sultanik, Olivier Deckmyn, Mattias Mandorfer, Marco Senzolo, Filippo Schepis, Dhiraj Tripathi, Juan Carlos Garcia Pagan, Dominique Thabut, a study by the Baveno Cooperation

Table of contents

Table S1……………………………………………………………………………2

Table S2……………………………………………………………………………3

Table S3……………………………………………………………………………4

Table S4……………………………………………………………………………5

Table S5……………………………………………………………………………6

Supplementary Material 1…………………………………………………………..7

Fig. S1……………………………………………………………………………...8

Fig. S2……………………………………………………………………………...9

Fig. S3……………………………………………………………………………...9

Fig. S4……………………………………………………………………………10

Table S1: starting date for EuroTIPS database according to center.

Pitié-Salpêtrière	4/12/2020
Barcelona	10/07/2020
Birmingham	20/01/2021
Toulouse	17/12/2020
Leuven	22/07/2022
Vienna	11/12/2020
Münster	16/01/2020
Padova	16/01/2020
Modena	22/01/2021

Table S2: baseline characteristics of patients with definite or probable AH

Characteristic	Definite AH n=80	Probable AH n=62	p
**Age**	52 (44 – 57)	55 (48 – 61)	0.03
**Male Sex, %**	67 (83)	52 (84)	0.99
**AVB as first decompensating event n (%)**	32 (40)	23 (37)	0.86
**Ascites at admission n (%)**	53 (66)	41 (66)	0.99
**HE at admission n (%)**	38 (48)	30 (48)	0.99
**Shock at admission n (%)**	40 (50)	21 (36)	0.18
**Infection at admission n (%)**	17 (23)	18 (36)	0.18
**Hemoglobin (g/L)**	8.1 (6.7 – 9.2)	7.9 (6.4 – 9.2)	0.21
**Platelets count (G/L)**	86 (48 – 121)	74 (50 – 97)	0.01
**INR**	2.20 (1.82 – 2.62)	2.20 (1.87 – 2.54)	0.84
**Bilirubin (μmol/L)**	131 (77-249)	103 (72-145)	0.08
**Albumin g/L**	26 (23-30)	25 (22 – 26)	0.05
**Creatinine μmol/L**	66 (54 – 101)	68 (66 – 71)	0.77
**MELD score**	23.7 (21.0 – 29.1)	23.3 (21.5 – 26.8)	0.74
**Child-Pugh class B/C n (%)**	9/71 (11/89)	8/54 (13/87)	0.80

Student's t test was used for group comparisons of normally distributed continuous variables. Group comparisons of categorical variables were performed using Chi-squared test. A p value <0.05 was considered significant. Abbreviations: AH, alcohol-related hepatitis; AVB, acute variceal bleeding; HE, hepatic encephalopathy; INR, international normalized ratio; PT, prothrombin time ratio; MELD, model for end stage liver disease;

Table S3: diagnosis of AH according to centers

	Definite AH n=80	Probable AH n=62	p
			0.01
Pitié-Salpêtrière (n=69)	61 (88%)	8 (12%)	
Spain (n=35)	9 (25%)	26 (75%)	
Toulouse (n=15)	6 (40%)	9 (60%)	
Leuven (n=5)	3 (60%)	2 (40%)	
Cluj (n=4)	0 (0%)	4 (100%)	
Others (n=14)	1 (7%)	13 (93%)	

Abbreviations: AH, alcohol-related hepatitis

Table S4: Baseline MELD score and bilirubin of patients included in randomized controlled trials on AVB or alcohol-related hepatitis

Study	*Clinical situation*	*MELD score*	*Bilirubin (μmol/L)*
Garcia-Pagan, NEJM 2010	*pTIPS in AVB*	*15.5*	*63*
Lv, Lancet GE 2019	*pTIPS in AVB*	*14*	*21*
Dunne, APT 2023	*pTIPS in AVB*	*15.5*	*68*
Louvet, JAMA 2023	*Alcohol-related hepatitis*	*25*	*272*
Thursz, NEJM 2015	*Alcohol-related hepatitis*	*21*	*290*
Our study	*pTIPS & Alcohol-related hepatitis*	*24*	*130*

pTIPS, preemptive TIPS; AVB; acute variceal bleeding

Table S5: Cumulative incidence of death during follow-up in patients treated with pTIPS or Endo+drugs (LT competing event)

Characteristic	Time 42	Time 90	Time 180	p-value[1]
LT				
Group				0.3
Endo+drugs	1.1% (0.09%, 5.4%)	1.1% (0.09%, 5.4%)	2.4% (0.45%, 7.6%)	
TIPS	6.9% (1.7%, 17%)	9.3% (2.9%, 20%)	9.3% (2.9%, 20%)	
death				
Group				0.2
Endo+drugs	30% (21%, 40%)	35% (25%, 45%)	38% (28%, 48%)	
TIPS	16% (7.0%, 29%)	16% (7.0%, 29%)	24% (12%, 37%)	

[1] Gray's Test

The Fine and Gray model was used and comparison between patients treated with pTIPS or Endo+drugs was performed using Gray's test.

Abbreviations: LT, Liver transplantation; pTIPS, preemptive transjugular intrahepatic portosystemic shunt;

Supplementary Material 1: Management of AH

Overall, 69/142 patients (49%) patients were treated with corticosteroids, without any difference between the 2 groups (55% (pTIPS) and 46% (Endo+drugs) groups, respectively, p=0.30, see Table A below). The proportion of patients who were treated with corticosteroids was significantly higher patients with definite AH compared to patients with probable AH (70 vs 21%, p<0.001). There was no significant difference in terms of MELD, bilirubin level or infection at admission. The median duration of corticosteroids treatment was similar between the TIPS and Endo+drugs groups (7 (7-28 days) vs 28 (13-28) days, p=0.09 and not different in patients with definite or probable AH (28 days (7-28 days), vs 7 (6.5-32 days, p=0.58)).

Table A: Management of AH

	Whole cohort n=142	pTIPS n=47	Endo+Drugs n=95	p
Corticosteroids n (%)	69 (49)	26 (55)	43 (46)	0.30
Number of patients treated with 28 days of corticosteroids (%)	30 (21)	11 (23)	19 (20)	0.90
Development of infection after corticosteroids therapy	37 (36)	13 (31)	24 (40)	0.30
Lille score calculation n (%)*	56 (81)	20 (77)	36 (84)	0.50
Lille score	0.36 (0.17-0.67)	0.44 (0.17-0.59)	0.35 (0.18-0.67)	0.90
Lille score >0.45	24 (43)	10 (50)	14 (39)	0.40

*in patients who were treated with corticosteroid therapy

Student's t test was used for group comparisons of normally distributed continuous variables. Group comparisons of categorical variables were performed using Chi-squared test. A p value <0.05 was considered significant.

Abbreviations: AH, alcohol-related hepatitis; pTIPS, preemptive transjugular intrahepatic portosystemic shunt

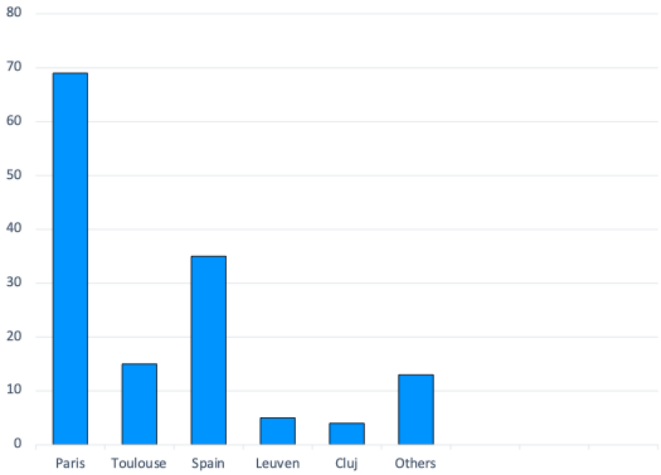

Supplementary Figure 1

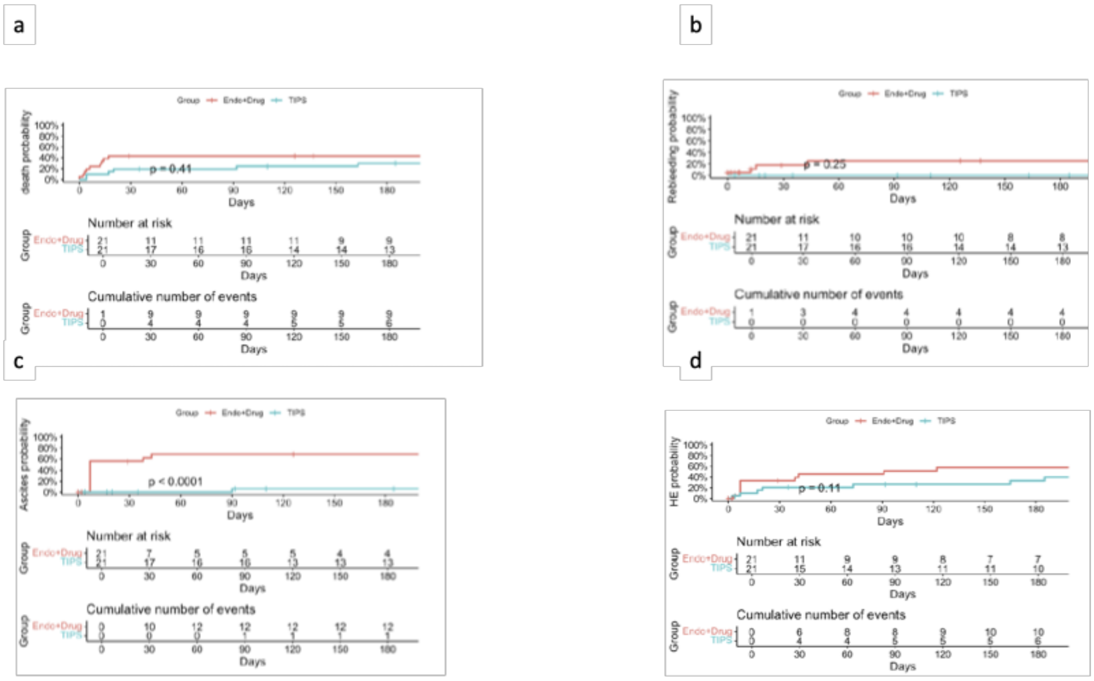

Supplementary Figure 2

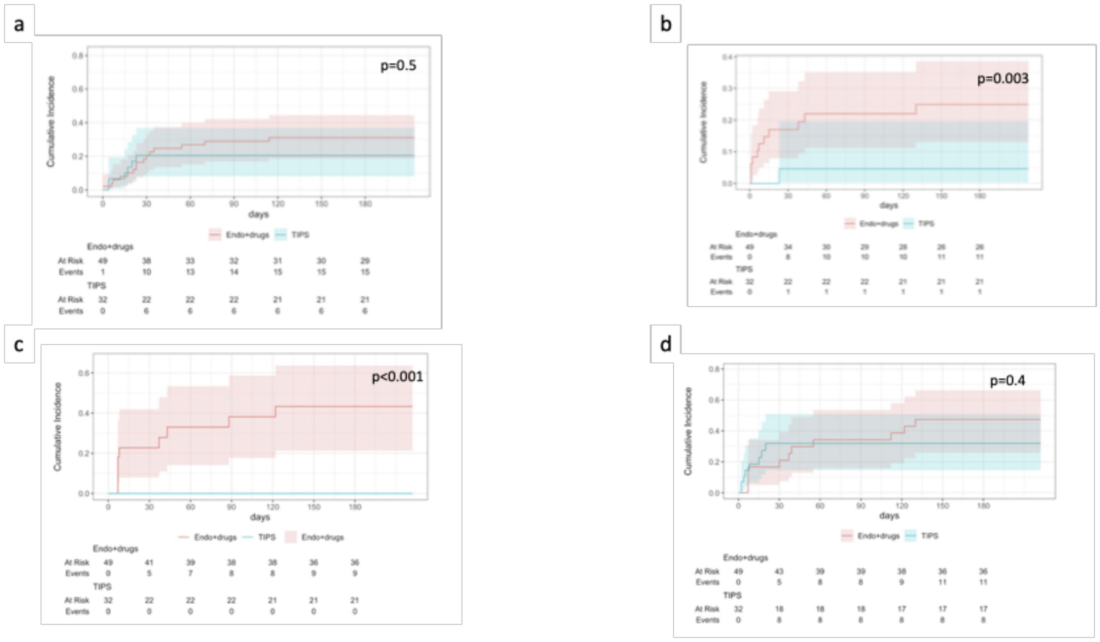

Supplementary Figure 3

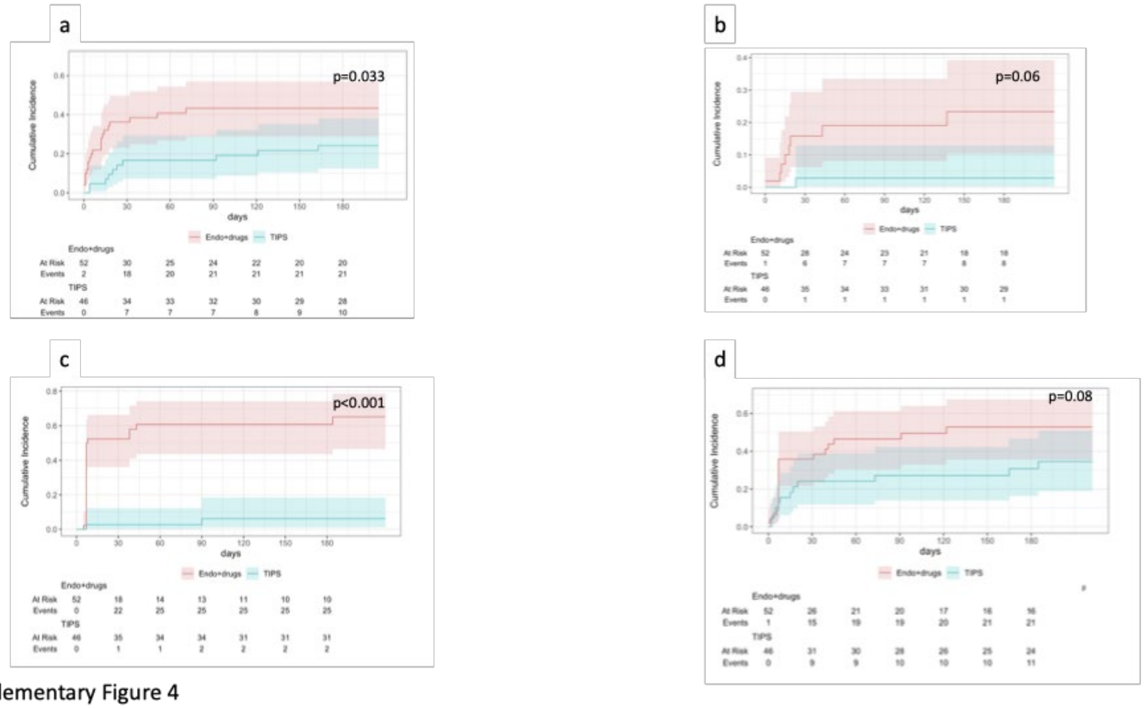

Supplementary Figure 4
