# Supplementary material for: Pre-emptive TIPS should be considered in high-risk patients with both acute variceal bleeding and severe alcohol-related hepatitis
Source: JHEP Rep. 2025 Sep 29;7(12):101611. doi: 10.1016/j.jhepr.2025.101611 (PMC12682117; doi:10.1016/j.jhepr.2025.101611)
Supplement: Multimedia component 4 [file mmc4.pdf]

JHEP|Reports

Pre-emptive TIPS should be considered in high-risk patients with both acute variceal bleeding and severe alcohol-related hepatitis

Authors

Marika Rudler, Virginia Hernández-Gea, Hélène Larrue, …, Dhiraj Tripathi, Juan Carlos Garcia Pagan, Dominique Thabut

Correspondence

marika.rudler@aphp.fr (M. Rudler).

Graphical abstract

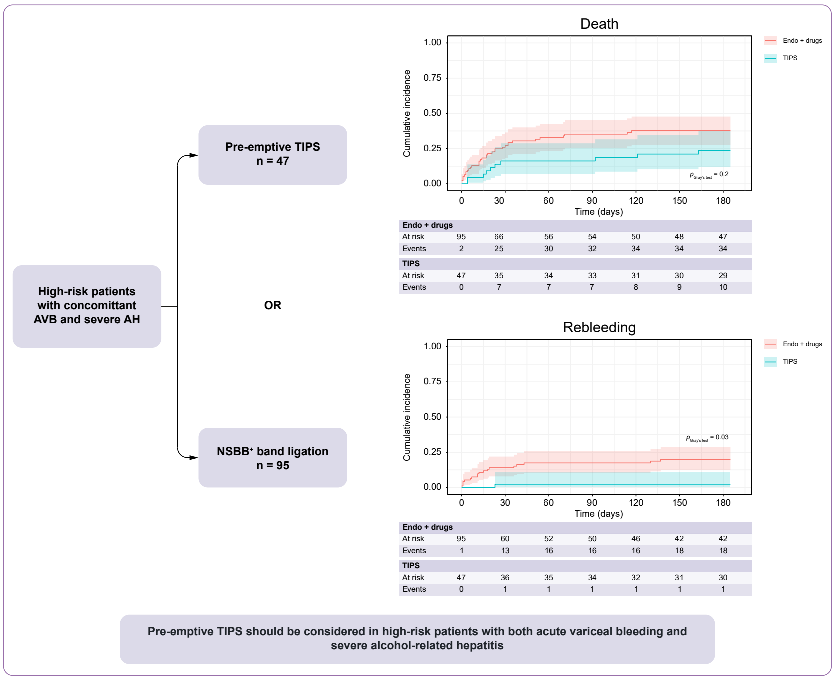

Highlights:

- Whether pTIPS should be performed in patients with concomitant severe alcohol-related hepatitis is matter of debate.
- pTIPS was associated with a lower rate of rebleeding and ascites compared with standard treatment.
- pTIPS was associated with a similar incidence of overt hepatic encephalopathy compared with standard treatment.
- pTIPS should not be contraindicated in high-risk patients with acute variceal bleeding and concomitant severe alcohol-related hepatitis.

Impact and implications:

Severe alcohol-related hepatitis and acute variceal bleeding may occur concomitantly, yet the role of pre-emptive transjugular intrahepatic portosystemic shunt (pTIPS) placement in this setting remains unclear. In this study, compared to standard of care, pTIPS treatment was associated with lower mortality, although this difference did not reach statistical significance, as well as a significantly reduced risk of rebleeding and recurrent ascites. These findings suggest that severe alcohol-related hepatitis should not be viewed as a contraindication to pTIPS placement when otherwise indicated, such as in patients with Child–Pugh B cirrhosis with a score greater than 7 and active bleeding, or Child–Pugh C10–13 disease.

https://doi.org/10.1016/j.jhepr.2025.101611

© 2025 The Author(s). Published by Elsevier B.V. on behalf of European Association for the Study of the Liver (EASL). This is an open access article under the CC BY license (http://creativecommons.org/licenses/by/4.0/).

Pre-emptive TIPS should be considered in high-risk patients with both acute variceal bleeding and severe alcohol-related hepatitis☆

Marika Rudler[1,2,3,*], Virginia Hernández-Gea[4], Hélène Larrue[5], Charlotte Bouzbib[1,2], Bogdan Procopet[6], Anna Baiges[4], Fanny Turon[4], Candido Villanueva[6], Agustin Albillos[7], Edilmar Alvarado Tapias[8], Lise Lott Gluud[9], Michael Praktiknjo[10], Joan Genesca[11], Meritxell Ventura-Cots[11], Ares Villagrasa[11], Susanna Rodrigues[12], Sarah Mouri[1,2], Álvaro Giráldez-Gallego[13], Helena Masnou Ridaura[14], Wim Laleman[10,15], Christophe Bureau[5], Marie-Angèle Robic[5], Lukas Hartl[16], Luis Tellez[7], Alexander Zipprich[17], Nuria Canete[18], Philippe Sultanik[1,2], Olivier Deckmyn[19], Mattias Mandorfer[16], Marco Senzolo[20], Filippo Schepis[21], Dhiraj Tripathi[22], Juan Carlos Garcia Pagan[4], Dominique Thabut[1,2,3], a study by the Baveno Cooperation

JHEP Reports **2025**. vol. 7 | 1–9

Check for updates

Background & Aims: Severe alcohol-related hepatitis (AH) and acute variceal bleeding (AVB) may occur simultaneously. The impact of a pre-emptive transjugular intrahepatic portosystemic shunt (pTIPS) in high-risk patients (patients with Child–Pugh (CP) B and active bleeding or CP C10–13 cirrhosis) with AVB and concomitant severe AH is unknown. The objective of the study was to compare the outcomes of severe AH in patients with high-risk AVB treated with pTIPS or endoscopic and drug treatment (Endo+drugs).

Methods: Patients were screened in four existing cohorts of patients with cirrhosis and AVB treated either with pTIPS or Endo +drugs. The inclusion criteria were AVB, high-risk patients, suspected severe AH (recent onset of jaundice, alcohol-related liver disease, absence of abstinence, model for end-stage liver disease score >20 and aspartate aminotransferase <500 UI/L). The primary endpoint was 42-day mortality, considering liver transplantation as a competing event. Secondary endpoints were rebleeding and further development of ascites or hepatic encephalopathy at 6 months.

Results: A total of 142 patients with AVB were included (pTIPS: n = 47, Endo+drugs: n = 95). Baseline characteristics (age 53, male sex 84%, model for end-stage liver disease score 23.4) were similar between the two groups. Overall, 56% had histologically proven AH. The 42-day mortality was 16% in the pTIPS group *vs.* 30% in the Endo+drugs group (p = 0.2). The cumulative incidence of rebleeding and ascites was significantly lower in the pTIPS group (2.8% *vs.* 24%, p = 0.026, and 6% *vs.* 52%, p <0.001, respectively), whereas hepatic encephalopathy occurrence was similar in the two groups (p = 0.2). Corticosteroid therapy was given in 55% and 46% of patients in the pTIPS and Endo+drugs groups, respectively (p = 0.3).

Conclusions: In severe AH, pTIPS is associated with better outcomes than Endo+drugs, and should not be contraindicated.

© 2025 The Author(s). Published by Elsevier B.V. on behalf of European Association for the Study of the Liver (EASL). This is an open access article under the CC BY license (http://creativecommons.org/licenses/by/4.0/).

Introduction

The prognosis of patients with cirrhosis and acute variceal bleeding (AVB) has dramatically improved with the placement of a pre-emptive transjugular intrahepatic portosystemic shunt (pTIPS) in patients at high risk of rebleeding.[1–4] Specifically, in patients with Child–Pugh B>7 cirrhosis and active bleeding or Child–Pugh C 10–13, a TIPS placed within 72 h of the onset of bleeding has been shown to decrease rebleeding and the occurrence of further decompensation, and to increase survival.[1–4] The benefit of pTIPS has also been demonstrated in patients with AVB presenting with acute-on-chronic liver failure (ACLF).[5] In Western studies published on pTIPS, excessive alcohol consumption accounts for 75% of the causes of liver disease.[1,6,7] In non-abstinent patients with AVB and jaundice, concomitant severe alcohol-related hepatitis (AH) may be suspected in a small subgroup. These patients may present with a recent onset of jaundice (<2 months), elevated transaminases, and a model for end-stage liver disease (MELD) score >20. The diagnosis of severe AH may be suspected after excluding other causes, such as shock. Severe AH is the phenotype of alcohol-related liver disease associated with the poorest prognosis, with a 2-month mortality of 25%.[8] This

☆ Given their role as co-editors, Virginia Hernández-Gea and associate editor, Mattias Mandorfer had no involvement in the peer-review of this article and had no access to information regarding its peer-review. Full responsibility for the editorial process for this article was delegated to the guest editor Annalisa Berzigotti.

* Corresponding author. Address: Hepatology Department, Sorbonne Université, Assistance Publique-Hôpitaux de Paris, La Pitié-Salpêtrière Hospital, 75013 Paris, France.
E-mail address: marika.rudler@aphp.fr (M. Rudler).
https://doi.org/10.1016/j.jhepr.2025.101611

depends on both the severity of the liver disease at baseline (as evaluated by a MELD score >20) and the response to corticosteroids (as evaluated by the Lille score[9]).

We previously showed in a retrospective study that, in patients with AVB and clinically suspected severe AH, liver biopsy was compatible with AH in 78% of cases.[10] Our findings also suggested that the prognosis of patients with concomitant severe AH and upper gastrointestinal bleeding treated with corticosteroids was similar to that of patients with AH without bleeding.[10] This result underscores the potential benefit of treating severe AH with corticosteroids, even in cases of acute bleeding. This point is important, as a recent episode of bleeding is an exclusion criterion in all randomized controlled trials evaluating new therapeutic options in severe AH.[11–13] Recently, Villagrasa *et al.*[14] found, in an ancillary study performed within a multicenter European cohort, that the phenotype of alcohol-related cirrhosis (with or without AH) had no impact on 1-year survival in patients with AVB,[14] although this study included patients with non-severe AH (median MELD score 19).

The impact of pTIPS in the specific subgroup of patients with concomitant severe AH and AVB at high risk of rebleeding has never been studied.

Hence, the aim of this study was to compare, within this multicenter international cohort, the prognosis of patients with AVB and AH treated with pTIPS or endoscopic band ligation and non-selective beta-blockers (NSBBs) (Endo+drugs) as secondary prophylaxis. This comparison used individual patient data from existing prospective and retrospective cohorts of AVB and severe AH.

Materials and methods

Patients

We established two groups of patients hospitalized for AVB and definite or probable severe AH. Patients were treated either with pTIPS (pTIPS group) within 72 h after stabilization of the index bleeding, or Endo+drugs (Endo+drugs group) for secondary prophylaxis of AVB. We used individual patient data from pre-existing prospective or retrospective databases (see details of the different cohorts below) (Hernández-Gea *et al.*,[6] Rudler *et al.*,[10] EuroTIPS group database). Inclusion and exclusion criteria were designed in accordance with the recommendations published for the non-invasive diagnosis of AH according to the National Institute on Alcohol Abuse and Alcoholism AH Consortia.[15] The inclusion criteria were all of the following: cirrhosis; AVB; alcohol-related cirrhosis; absence of abstinence; jaundice; bilirubin >3 mg/dl; aspartate aminotransferase (AST) >50 U/L; MELD score >20; and high risk of rebleeding, defined by a Child–Pugh score B >7 with active bleeding at endoscopy, or Child–Pugh C10–13. Exclusion criteria were: previous liver transplantation (LT); other concomitant causes of liver disease; AST >500 U/L; and absence of AH on liver biopsy (when performed).

To build the pTIPS group, we included patients from three existing cohorts: (1) patients selected from the EuroTIPS database, a prospective cohort encompassing all consecutive patients treated with TIPS in nine European centers (Paris, Toulouse, Barcelona, Vienna, Padova, Modena, Birmingham, Münster, Leuven). Data collection started in 2020 for the majority of centers (Table S1); (2) patients were screened from the Paris (Pitié-Salpêtrière hospital) prospective TIPS database, which collected data from all consecutive patients treated with TIPS between 2018 and 2020 (before the start of the EuroTIPS database); (3) patients selected from the multicenter prospective observational study involving patients with AVB in 34 referral centers across Europe and Canada (International Variceal Bleeding Observational Study Group and Baveno Cooperation), included between October 2011 and May 2015. While this study included patients treated with either pTIPS or Endo+drugs,[6] only patients treated with pTIPS were considered for this group.

To build the Endo+drugs group, we selected patients from two existing cohorts: (1) patients selected from the multicenter prospective observational study involving patients with AVB in 34 referral centers across Europe and Canada (International Variceal Bleeding Observational Study Group and Baveno Cooperation), included between October 2011 and May 2015. While this study included patients treated with either pTIPS or Endo+drugs,[6] only patients treated with Endo +drugs were considered for this group; (2) patients from the Paris retrospective database that was previously published.[10] This cohort included patients with histologically proven AH, with or without upper gastrointestinal bleeding, hospitalized in our Hepatology intensive care unit between September 2005 and March 2011. We selected patients who presented with concomitant AVB and severe AH. As pTIPS was systematically implemented in this center in March 2011, we only included patients before March 2011.

Management of AVB

All patients were treated according to the Baveno recommendations in effect during the year of hospitalization for AVB:[16–19] they all received vasoactive drugs, antibiotic therapy, and endoscopic band ligation at the time of bleeding. Notably, patients with gastric varices were not excluded. In the Endo+drugs group, NSBB and repeated band ligation were performed regularly until variceal eradication. In the pTIPS group, TIPS was placed within 72 h of stabilization, according to each center's protocol. Polytetrafluoroethylene-covered stents (Viatorr®, TIPS endoprosthesis, Gore, Flagstaff, AZ, United States) were used in all centers. The technique of TIPS placement was not modified when AH was suspected.

Management of severe AH

The management of severe AH was determined by each center's protocol. Notably, some centers treated patients with corticosteroids systematically, others never did, and some decided on a case-by-case basis.

Data collection

Patients were included with the day of index bleeding defined as day 0. The clinical and laboratory data collected at admission were: sex, age, previous history of liver decompensation (ascites, hepatic encephalopathy [HE], AVB), presence of ascites, HE, hemoglobin, platelets, leukocytes, prothrombin time (PT), bilirubin, lactate, creatinine, albumin, serum sodium, international normalized ratio (INR), AST, alanine aminotransferase, gamma-glutamyl transferase, liver biopsy (if available),

treatment with corticosteroids (and duration of corticosteroid therapy, if applicable), and efficacy as determined by the Lille score (lillemodel.com[9]).

Definitions

AH was classified as definite if liver biopsy showed necrosis, ballooning, neutrophil infiltration, and Mallory bodies. AH was classified as probable if liver biopsy was unavailable and all non-invasive inclusion criteria were met.[15] AH was excluded in cases where liver biopsy did not show all histological signs of AH, even in patients meeting the non-invasive diagnostic criteria for AH. Patients were considered non-responders to corticosteroids if the Lille score at day 7 was >0.45. Regarding infection, it was defined as positivity for ascites with increased neutrophil count >250/mm^3, a positive blood or urine culture, or clinically evident pulmonary infection with abnormal chest X-ray. We differentiated infection at admission (infection diagnosed on the day of admission) and development of infection (infection diagnosed after the first day of corticosteroid therapy). Further decompensation included rebleeding, HE, or ascites. Rebleeding was defined as recurrent melena or hematemesis, resulting in hospital admission, blood transfusion, a 3 g/dl drop in hemoglobin, or death within 6 weeks after admission.[17]

Outcomes

The primary endpoint was 42-day mortality, considering LT as a competing event. Secondary endpoints were: 6-month mortality; further decompensation (rebleeding, ascites, or HE), considering LT and death as competing events at 6 months; further development of infection; and 6-month mortality stratified by Lille score.

Ethics

The study protocol for patients included in the EuroTIPS database and the International Variceal Bleeding Observational Study Group and Baveno Cooperation were approved by the ethics committee of each participating center. For the remaining patients, data collection was approved by the research ethics committee of Sorbonne University (CER-2022-074).

Patient and public involvement

Patients and members of the public were not involved in research.

Number of patients calculation

In the previous study of our group on severe AH and AVB treated with Endo+drugs, the 6-month mortality rate was 26%.[10] We hypothesized that the 6-month mortality rate in the pTIPS group would be approximately 12.5%.[1] With an alpha risk of 5% and a beta risk of 20%, the required sample size was 260 patients.

Statistical analyses

Data were presented by means and standard deviations/median and IQRs for normally/non-normally distributed continuous variables, and frequencies and percentages for categorical data. Characteristics of patients were compared using X^2 (for categorical variables) and independent-samples *t*/Wilcoxon test (for normally/non-normally distributed continuous variables). Survival rates were calculated using the Kaplan–Meier method, and compared using the log-rank test. Hazard ratios and 95% CIs were calculated using Cox proportional hazard ratio survival analysis. We performed Gray's test to compare mortality (with LT as competing event) or any further decompensating event (with LT and death as competing events) between different groups. Univariate analyses used all available variables. Selected variables that associated with the outcome in the univariate analysis were incorporated into the multivariate analysis, added to the group of treatment (pTIPS or Endo+drugs). The maximal number of variables into each multivariate model was defined as N event/10. Proportional hazards assumption was checked based on smoothed plots of Schoenfeld residuals. To confirm our results, we performed a propensity matched analysis of the patients receiving pTIPS and those without pTIPS. We also performed sensitivity analysis in two subgroups of patients: with definite severe AH and with probable severe AH.

All statistical analyses were performed using RStudio software (2023.12.0+369, RStudio, Inc., Boston, MA, USA), Posit team (2023). A two-tailed *p* value <0.05 was considered as significant.

Results

Patients

Overall, 166 patients meeting non-invasive criteria were screened: 52 patients in the pTIPS group and 114 in the Endo+drugs group (Fig. 1). Liver biopsy was performed in 104/166 (62.5%) patients (37/52 [71.1%] patients in the pTIPS group *vs.* 67/114 [58.7%] in the Endo+drugs group, *p* = 0.03). Among those biopsied, AH was histologically confirmed and classified as definite in 80/104 patients (76.9%): 32/37 (86.5%) in the pTIPS group and 48/67 (71.6%) in the Endo+drugs group (*p* = 0.51); 62 patients had probable AH. The baseline characteristics of patients with definite or probable AH are detailed in Table S2. There was heterogeneity among centers regarding the rates of definite AH (from 0% to 88%, *p* = 0.01, Table S3).

AH was ruled out in five patients in the pTIPS group and in 19 patients in the Endo+drugs group; these patients were excluded from the analysis. Altogether, 142 patients were included in the current study: 47 in the pTIPS group and 95 in the Endo+drugs group (Fig. 1). The number of patients included in each participating center is detailed in Fig. S1.

Baseline characteristics of the overall cohort are presented in Table 1: 119/142 (84%) patients were male, with a median age of 53 (48–63). Median bilirubin was 105 μmol/L (69–187), and the median MELD score was 23.4 (21.0–27.9). At admission, 94/142 (66%) patients presented with ascites, 68/142 (48%) with HE, 61/142 (45%) with shock, and 35/142 (25%) with infection. There were no statistically significant differences between the pTIPS and Endo+drugs groups, except for platelet count, which was lower in the pTIPS group (72 *vs.* 89 G/L, *p* = 0.02), and bilirubin level, which was higher in the pTIPS group (130 *vs.* 97 μmol/L, *p* = 0.03). In the pTIPS group, median dilatation of the stent was 8 mm (8–8). AH was classified as definite in 68% of patients in the pTIPS group *vs.* 51% of patients in the Endo+drugs group (*p* = 0.04). Detailed data

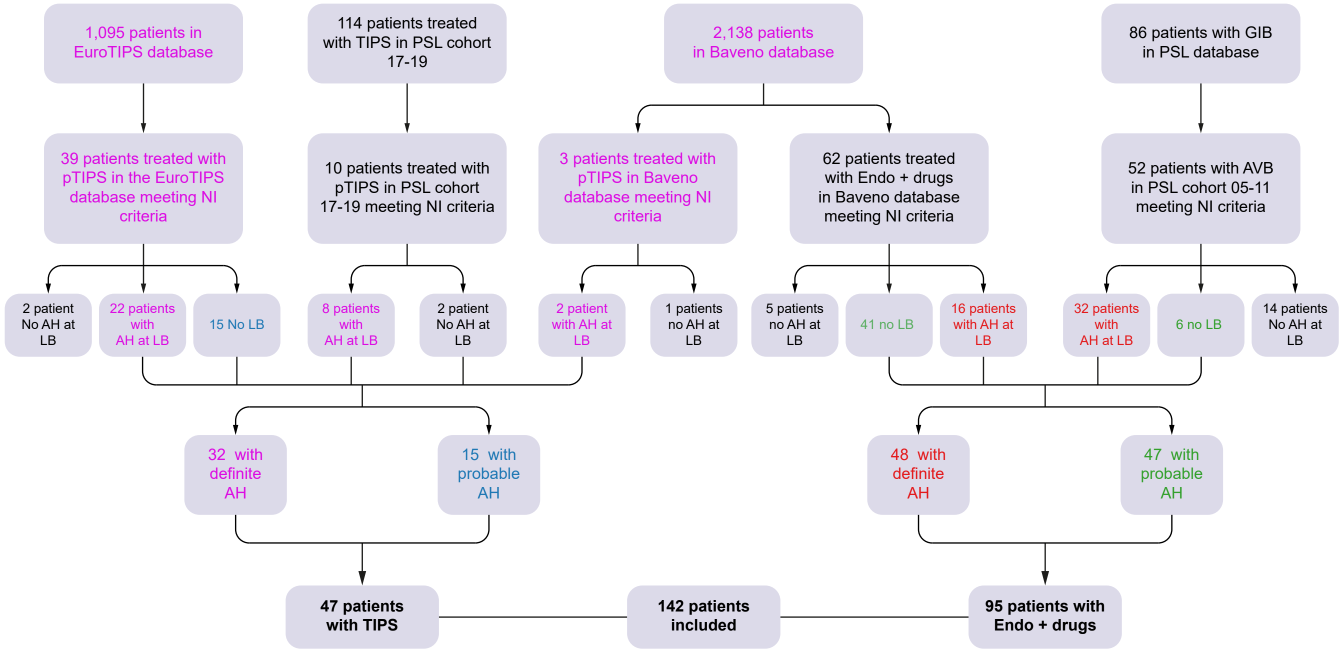

Fig. 1. Flowchart of patients that were included in the study. Forty-seven patients were included in the pTIPS group (38 from the EuroTIPS group and nine from the prospective database of Paris) and 95 patients in the Endo+drugs group (61 in the Baveno variceal bleeding database and 34 in the retrospective database of Paris). AH, alcohol-related hepatitis; GIB, gastrointestinal bleeding; LB, liver biopsy; NI, non-invasive; TIPS, transjugular intrahepatic portosystemic shunt.

regarding AH management are provided in Supplementary material 1. A table including a qualitative comparison of the baseline liver function of patients included in pTIPS studies and severe AH studies is provided (Table S4).

Outcomes

The median follow-up was 205 days (IQR 21–433).

Mortality and LT at day 42 and 6 months

After 42 days of follow-up, 34 patients had died and four patients had undergone LT, with no significant difference between the pTIPS and Endo+drugs groups (Table S5). Considering LT as a competing event, there was no significant difference in 42-day mortality between the two groups (16% [95% CI, 7.0–29%] *vs.* 30% [95% CI, 21–40%], $p = 0.2$) (Fig. 2). Table 2 shows the univariate and multivariate analyses of factors associated with 42-day mortality; the only independent factor that remained significantly associated with higher 42-day mortality was MELD score (subdistribution hazard ratio [sHR] 1.06 [95% CI, 1.01–1.11], $p = 0.01$). The 6-month mortality and LT rates were also not significantly different in patients treated with pTIPS or Endo+drugs (24% [95% CI, 12–37%] *vs.* 38% [95% CI, 28–48%], $p = 0.2$, and 9.3% [95% CI, 2.9–20.0%] *vs.* 2.4% [95% CI, 0.45–7.6%], $p = 0.3$ for mortality and LT, respectively, Table S4).

Further decompensation at 6 months

Further decompensation was evaluated, considering LT and death as competing events. The cumulative incidence of rebleeding was significantly lower in the pTIPS group (2.8% [95% CI, 0.2–13%] *vs.* 24% [95% CI, 15–34%], $p = 0.03$, Fig. 3A), as was the cumulative incidence of ascites (6% [95% CI, 1–18%] *vs.* 52% [95% CI, 37–64%], $p < 0.001$, Fig. 3B). Conversely, the cumulative incidence of HE was similar between the two groups (30% [95% CI, 16–46%] *vs.* 47% [95% CI, 33–60%], $p = 0.2$, Fig. 3C).

Further development of infection

Further development of infection was diagnosed in 36 patients (35%) (data not available for 39 patients). The proportion of patients with further development of infection was not significantly different between the groups (29.7% *vs.* 40.0%, $p = 0.28$), nor in patients with or without corticosteroids (30.5% *vs.* 37.0%, $p = 0.64$).

Lille score calculation and performance in the prediction of 6-month survival

Overall, 69 patients received corticosteroids (Supplementary material 1). The Lille score was calculable in 56/69 (81%) patients who received corticosteroids (20 [77%] patients in the pTIPS group and 36 [84%] in the Endo+drugs group, $p = 0.5$). The median Lille score was 0.36 (0.17–0.68) in the overall cohort and not significantly different between the pTIPS (0.44 [0.17–0.59]) and Endo+drugs (0.35 [0.18–0.67], $p = 0.90$) groups. The proportion of non-responders according to the Lille score was similar between the pTIPS and Endo+drugs groups (50% *vs.* 39%, $p = 0.40$), as well as when comparing patients with probable or definite AH (57.1% *vs.* 42.5%, $p = 0.69$).

The 6-month survival rate was significantly higher in patients with a Lille score <0.45 who were treated with Endo+drugs (72.7% *vs.* 35.7%, $p = 0.04$), but not in patients treated with pTIPS (87% *vs.* 80%, $p = 0.54$) (Fig. 4A and B).

Table 1. Baseline characteristics of the 142 patients included in the study, 47 in the pTIPS group and 95 in the Endo+drugs group.

Characteristic	Whole cohort n = 142	pTIPS group n = 47	Endo+drugs n = 95	*p* value
Age	53 (48–63)	53 (48–60)	52 (47–61)	0.44
Male sex, %	119 (84)	39 (83)	80 (84)	0.90
AVB as first decompensating event n (%)	55 (39)	17 (36)	38 (40)	0.80
Ascites at admission n (%)	94 (66)	66 (69)	28 (60)	0.60
HE at admission n (%)	68 (48)	52 (55)	16 (35)	0.60
Shock at admission n (%)	61 (45)	49 (54)	12 (26)	0.05
Infection at admission n (%)	35 (25)	5 (11)	30 (32)	0.06
Antibiotherapy	138 (79)	47 (100)	91 (96)	0.90
Hemoglobin (g/L)	8.1 (6.8–9.2)	8.0 (6.2–9.2)	8.2 (7.3–9.1)	0.30
Platelets count (G/L)	78 (160–105)	72 (52–97)	89 (68–120)	0.02
INR	2.2 (1.85–2.57)	2.26 (1.93–2.73)	2.0 (1.70–2.44)	0.20
Bilirubin (µmol/L)	105 (69–187)	130 (77–195)	97 (66–173)	0.03
Albumin g/L	25 (22–28)	26 (22–30)	25 (22–27)	0.14
PT, %	39 (31–45)	38 (30–47)	41 (31–45)	0.90
Creatinine µmol/L	67 (55–94)	66 (54–90)	74 (60–131)	0.30
MELD score	23.4 (21.0–27.9)	24.0 (21.8–28.0)	23.4 (21.0–27.9)	0.90
Child–Pugh class B/C n (%)	17/125 (12/88)	4/43 (9/91)	13/82 (14/86)	0.70
Definite AH n (%)	80 (56)	32 (68)	48 (51)	0.04
Probable AH n (%)	62 (44)	15 (32)	47 (49)	0.04

Student's *t* test was used for group comparisons of normally distributed continuous variables. Group comparisons of categorical variables were performed using the X^2 test. A *p* value <0.05 was considered significant. AH, alcohol-related hepatitis; AVB, acute variceal bleeding; HE, hepatic encephalopathy; INR, international normalized ratio; MELD, model for end-stage liver disease; PT, prothrombin time ratio.

Sensitivity analyses

A propensity score-matched sensitivity analysis based on bilirubin level, shock at admission, platelet count, and definite AH was performed. The outcomes regarding mortality, rebleeding, and HE were similar between the two groups. However, the cumulative incidence of ascites was significantly higher in patients treated with Endo+drugs (Fig. S2).

After excluding patients with suspected AH, a sensitivity analysis on patients with only definite AH was performed. Similar results were obtained for patients treated with pTIPS or Endo+drugs regarding LT-free survival, rebleeding, ascites, and HE at 6 months (Fig. S3).

After excluding patients who were retrospectively included, a sensitivity analysis on patients who were prospectively included was performed. In the pTIPS group, mortality and the cumulative incidence of ascites were significantly lower at 6 months (Fig. S4). The cumulative incidence of rebleeding and HE were not significantly lower (p = 0.06 and p = 0.08, respectively).

Discussion

In this retrospective study of patients with suspected severe AH and AVB who were candidates for pTIPS, we found that, as in patients without suspected severe AH, pTIPS improved outcomes by decreasing the rates of rebleeding and ascites without increasing the rate of HE. Although there was a noticeable trend, survival was not significantly increased in patients who underwent pTIPS; however, we hypothesize that this could be a result of a type II error, as the study included only 47 patients treated with pTIPS and 95 with Endo+drugs. Our results support the idea that pTIPS should not be contraindicated in high-risk patients with concomitant AVB and AH. To our knowledge, this finding has not been reported previously: the study by Villagrasa *et al.*[14] included patients with non-severe AH, and very few of those patients were treated with pTIPS. Furthermore, the Baveno bleeding cohort was not designed to focus on AH or alcohol-related liver disease; therefore, data on concomitant AH diagnoses were not recorded, and traditional severity scores (*e.g.* Maddrey Discriminant Function) and treatments for AH were not registered.

We believe that this question is of interest. Most European studies have shown that pTIPS improves survival in high-risk patients with AVB,[1–4] many of whom had alcohol-related cirrhosis. In the randomized controlled trial published in 2010,[1] 70% of patients had alcohol-related cirrhosis, <50% were abstinent, the median bilirubin was >3 mg/dl, and the median MELD score was between 15 and 16, suggesting that some of these patients had a MELD score >20 and therefore met the non-invasive criteria for severe AH.

Besides our results, interesting questions remain unanswered, such as the role of corticosteroid therapy after pTIPS and the evaluation of its efficacy. Only 49% of patients received corticosteroids in the present study, and their use was not associated with a better prognosis at 1 month. This could be related to the limited power of our study, the low number of patients treated with corticosteroids, but also to the interaction with another therapeutic strategy (pTIPS), which also influences survival. Among patients who received corticosteroids in addition to pTIPS, the Lille model did not accurately predict short- or long-term survival. The Lille model includes early changes in bilirubin, a biological parameter that may be influenced by TIPS placement for either liver failure or hemolysis. Further prospective studies are warranted, and a new model needs to be developed for this setting.

We acknowledge that our study has several limitations, the first being its retrospective design. Second, the relatively small number of patients in the pTIPS group limited our ability to demonstrate a statistically significant difference in survival between the two groups, likely because of insufficient statistical power. The patient numbers are relatively low overall (38/1095 and 61/2138 in the two cohorts of pTIPS and Endo+drugs) in our series, which may be explained by limited access for these patients to tertiary care centers that often select patients eligible for LT.[20] Third, the accuracy of the AH

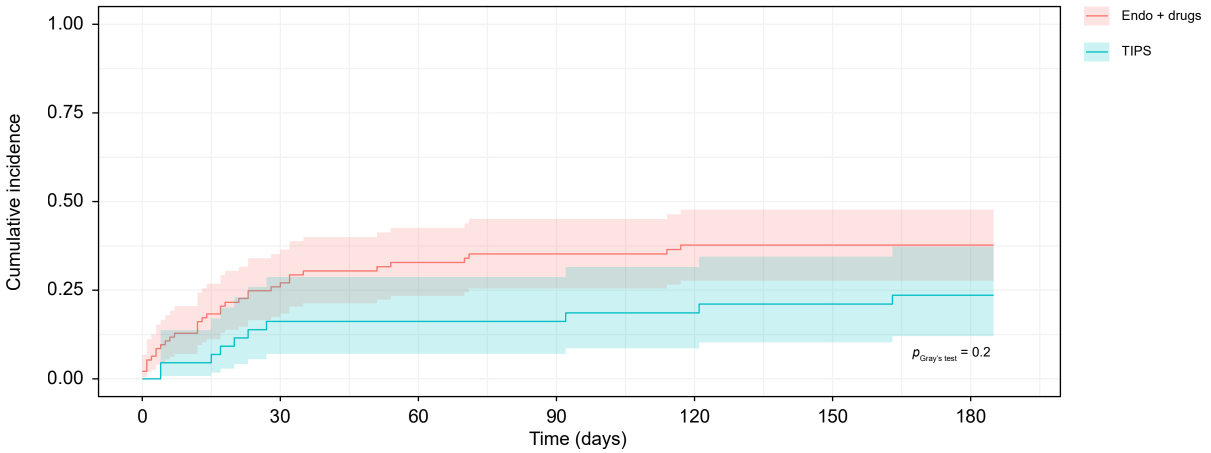

**Endo + drugs**							
At risk	95	66	56	54	50	48	47
Events	2	25	30	32	34	34	34
**TIPS**							
At risk	47	35	34	33	31	30	29
Events	0	7	7	7	8	9	10

Fig. 2. Cumulative incidence of mortality in patients treated with TIPS or Endo+drugs considering liver transplantation as a competing event. Forty-two-day mortality was not significantly different in patients treated with TIPS or Endo+drugs (16% [7.0%, 29%] *vs.* 30% [21%, 40%], *p* = 0.2). The Fine and Gray model was used and comparison between TIPS and Endo+drugs patients was performed using Gray's test. TIPS, pre-emptive transjugular intrahepatic portosystemic shunt.

diagnosis in our study is questionable: the most reliable clinical sign of AH is 'recent jaundice' within 3 months before admission, but the medical history of these patients is often incomplete. Moreover, few patients underwent liver biopsy to confirm the diagnosis, which is considered gold standard for the diagnosis of AH. Fourth, the small number of patients treated with corticosteroids prevented us from evaluating the performance of the Lille model in AVB for the prediction of 6-month survival. Finally, the proportion of patients who achieved abstinence could not be evaluated in this study. The impact of alcohol consumption relapse on long-term (>1 year) survival is well documented,[21] but it may have influenced 6-month survival.

Despite these limitations, the strength of the current study relies in its unique cohort of patients with concomitant AVB and AH, treated with either pTIPS or Endo+drugs. Because of the increasing adoption of pTIPS in both academic and non-academic centers, collecting more extensive data regarding high-risk patients with AVB treated with Endo+drugs as secondary prophylaxis (which was the previous standard of care), is very difficult. Moreover, we would like to emphasize that pTIPS was not deleterious in our study, suggesting that AH is not a condition in which pTIPS should be contra-indicated. Accumulating data on the management of AVB with pTIPS should encourage the systematic application of this policy, even in the subgroup of patients with the most severe presentations (with severe jaundice (3), five ACLF (5), andwith HE at admission).[22]

In conclusion, pTIPS is not contraindicated in patients with concomitant AVB and severe AH. Severe AH is likely under-diagnosed and undertreated in high-risk patients with AVB. In these patients, a liver biopsy should be considered. Further prospective studies are needed to better define the optimal management strategies for this population.

Table 2. Factors associated with 42-day mortality or LT in univariate and multivariate analysis.

	42-day death or LT UV analysis		42-day mortality or LT MV analysis	
**Factor**	**sHR (CI 95%)**	***p* value**	**sHR (CI 95%)**	***p* value**
Male sex	0.59 (0.28–1.25)	0.20	–	
Shock at admission	1.32 (0.71–2.45)	0.40	–	
Ascites at admission	0.91 (0.43–1.93)	0.80	–	
Infection at admission	1.42 (0.69–1.97)	0.30	–	
MELD score	**1.06 (1.01–1.10)**	**0.01**	**1.06 (1.01–1.11)**	**0.01**
pTIPS	1.00 (0.54–1.87)	0.90	–	
Corticosteroid therapy	1.19 (0.75–1.89)	0.50	1.6 (0.95–2.7)	0.07

Bolded values indicate statistically significant differences (*p* <0.05) (Fine and Gray models). LT, liver transplantation; MELD, model for end-stage liver disease; MV, multivariate analysis; pTIPS, pre-emptive portosystemic shunt; sHR, subdistribution hazard ratio; UV, univariate analysis.

A

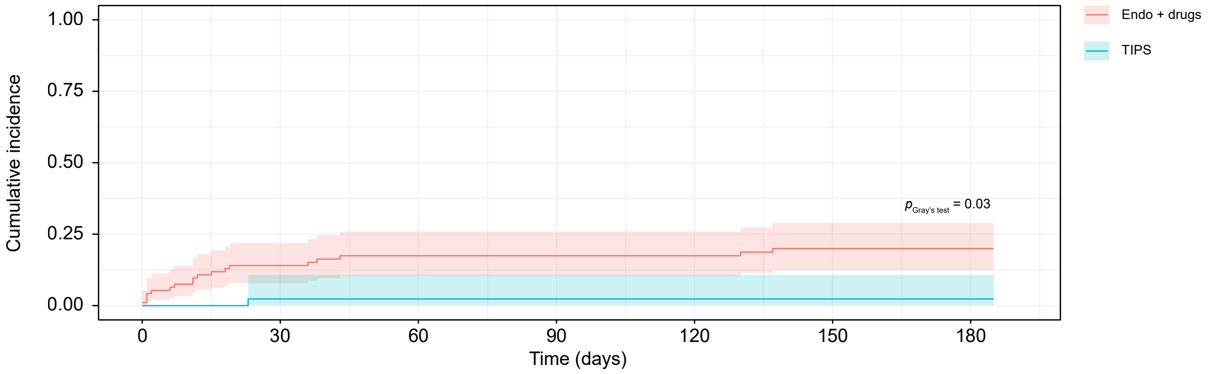

**Endo + drugs**							
At risk	95	60	52	50	46	42	42
Events	1	13	16	16	16	18	18
**TIPS**							
At risk	47	36	35	34	32	31	30
Events	0	1	1	1	1	1	1

B

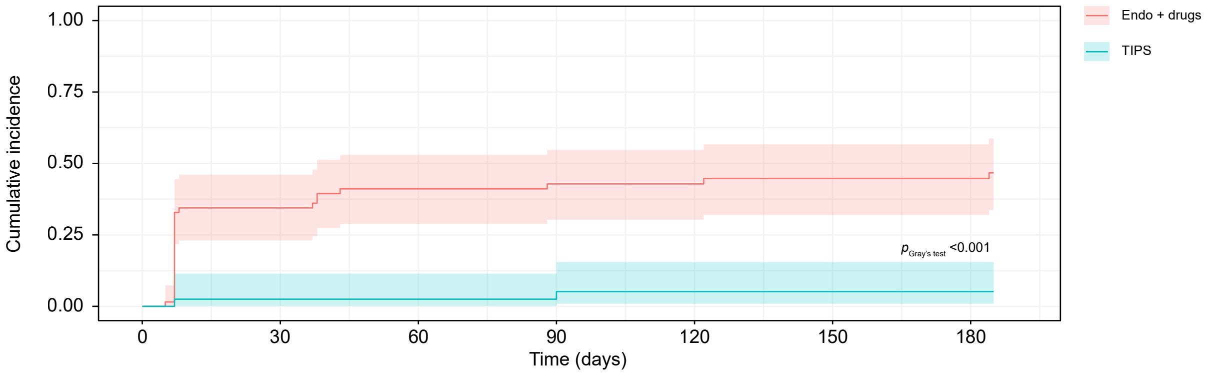

**Endo + drugs**							
At risk	95	61	56	54	52	50	50
Events	0	22	26	27	27	28	28
**TIPS**							
At risk	47	36	35	35	32	32	32
Events	0	1	1	2	2	2	2

C

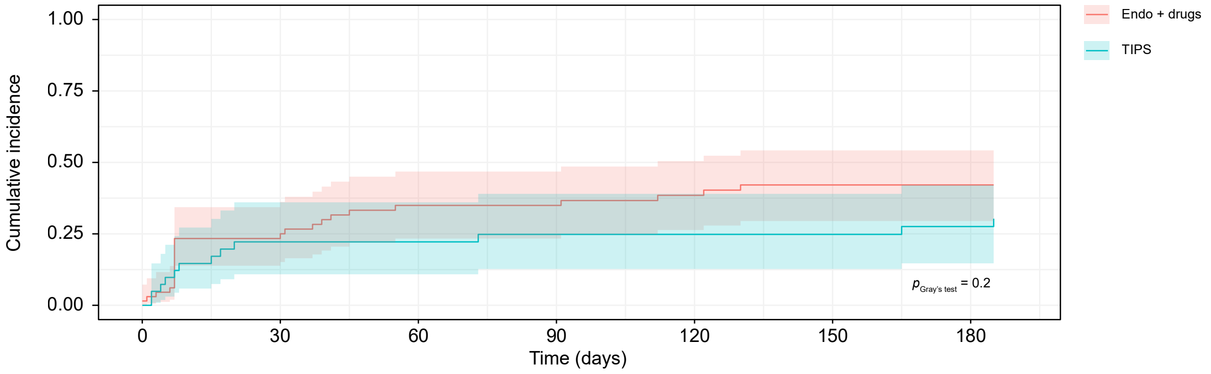

**Endo + drugs**							
At risk	95	69	61	60	56	54	54
Events	1	16	22	22	24	26	26
**TIPS**							
At risk	47	32	31	29	27	26	25
Events	0	9	9	10	10	10	11

Fig. 3. Cumulative incidence of rebleeding, ascites, and HE at 6 months in patients treated with pTIPS or Endo+drugs considering death and liver transplantation as competing events. The cumulative incidence of rebleeding and ascites was significantly lower in the pTIPS group (A and B), whereas the cumulative incidence of HE was similar between the two groups (C). The Fine and Gray model was used and comparison between patients who received pTIPS and Endo+drugs was performed using Gray's test. HE, hepatic encephalopathy; TIPS, transjugular intrahepatic portosystemic shunt.

A

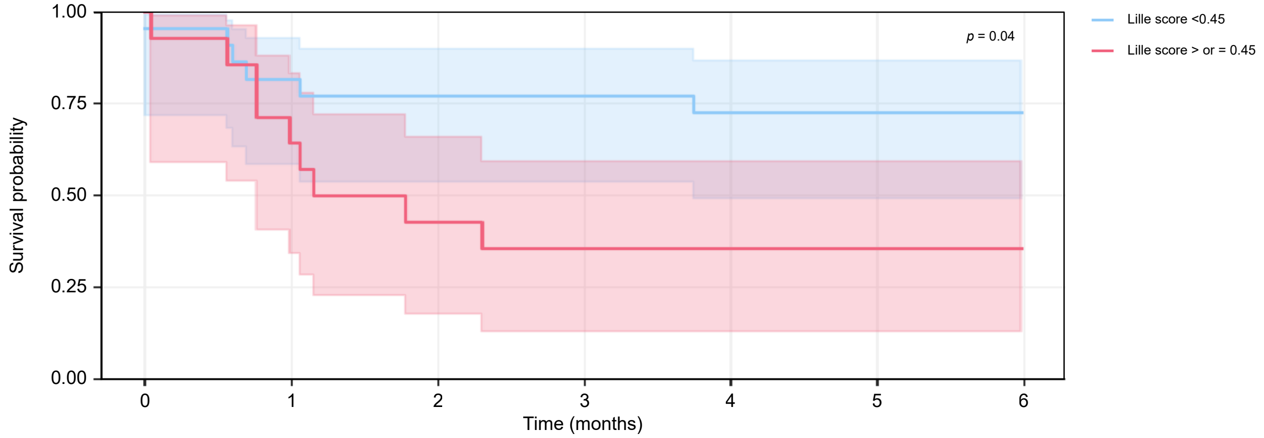

No							
At risk	21	18	17	17	16	16	0
Censored	0	0	0	0	0	0	16
Events	1	4	5	5	6	6	6

Yes							
At risk	14	9	6	5	5	5	0
Censored	0	0	0	0	0	0	5
Events	0	5	8	9	9	9	9

B

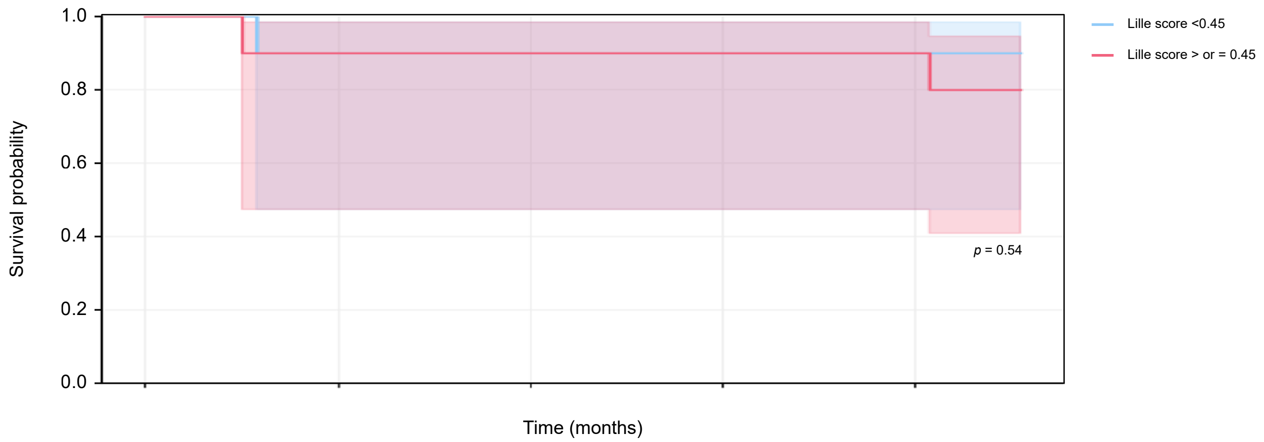

No						
At risk	10	9	9	9	9	0
Censored	0	0	0	0	0	9
Events	0	1	1	1	1	1

Yes						
At risk	10	9	9	9	9	0
Censored	0	0	0	0	0	8
Events	0	1	1	1	1	2

Fig. 4. Six-month survival in patients treated with Endo+drugs (A) or pTIPS (B) in responders and non-responders according to the Lille score. The 6-month survival was significantly higher in patients with a Lille score <0.45 in patients treated with Endo+drugs (72.7% *vs.* 35.7%, p = 0.04), but not in patients treated with pTIPS (87% *vs.* 80%, p = 0.54). pTIPS, pre-emptive transjugular intrahepatic portosystemic shunt.

Affiliations

[1]*AP-HP, Sorbonne Université, Liver Intensive Care Unit, Hepatogastroenterology Department, La Pitié-Salpêtrière Hospital, Paris, France;* [2]*INSERM UMR_S 938, Centre de recherche Saint-Antoine, Maladies métaboliques, biliaires et fibro-inflammatoire du foie, Institute of Cardiometabolism and Nutrition (ICAN), Paris, France;* [3]*Brain-Liver Pitié-Salpêtrière Study group (BLIPS), Paris, France;* [4]*Liver Unit, Hospital Clínic, Institut de Investigacions Biomèdiques August Pi i Sunyer (IDIBAPS), Barcelona, Spain;* [5]*Service d'Hépatologie Hôpital Rangueil CHU Toulouse et Université de Toulouse, Toulouse, France;* [6]*Department of Gastroenterology, Hospital de Sant Pau and CIBERehd, Barcelona, Spain;* [7]*Department of Gastroenterology and Hepatology, Hospital Universitario Ramón y Cajal, Instituto Ramón y Cajal de Investigación Sanitaria (IRYCIS), Universidad de Alcalá, Madrid, Spain;* [8]*Department of Gastroenterology, Hospital Santa Creu i Sant Pau, Autonomous University of Barcelona, Department of Medicine, Biomedical Research Institute Sant Pau (IIB Sant Pau), Barcelona, Spain;* [9]*Gastro Unit, Copenhagen University Hospital, Hvidovre, Denmark;* [10]*Department of Medicine I, University Hospital Bonn, Bonn, Germany;* [11]*Liver Unit, Digestive Diseases Area,*

Vall d'Hebron University Hospital, Vall d'Hebron Institute of Research (VHIR), Vall d'Hebron Barcelona Hospital Campus, Autonomous University of Barcelona, Barcelona, Spain; [12]Gastroenterology and Hepatology Department, Centro Hospitalar São João, Porto, Portugal; [13]UCM Digestive Diseases and CIBEREHD, University Hospital Virgen del Rocío, Institute of Biomedicine of Seville (CSIC/HUVR/US), University of Seville, Seville, Spain; [14]Division of Gastroenterology and Hepatology, IRCCS Ca' Granda Maggiore Hospital Foundation, University of Milan, Milan, Italy; [15]Department of Gastroenterology and Hepatology, Division of Liver & Biliopancreatic disorders and Liver Transplantation, University Hospitals Leuven, KU Leuven, Leuven, Belgium; [16]Department of Medicine III, Division of Gastroenterology and Hepatology, Medical University of Vienna, Vienna, Austria; [17]First Department of Internal Medicine, Martin Luther University Halle-Wittenberg, Halle (Saale), Germany; [18]Liver Section, Gastroenterology Department, Hospital del Mar, Universitat Autònoma de Barcelona, Barcelona, Spain; [19]BioPredictive, Paris, France; [20]Unit of Vascular Liver Diseases and Treatment of Portal Hypertension, Gastroenterology, Department of Surgery, Oncology and Gastroenterology, University Hospital of Padua, Padua, Italy; [21]Severe Liver Diseases Departmental Unit (M.E.C.), AOU Policlinico of Modena, University of Modena and Reggio Emilia, Modena, Italy; [22]Liver Unit, University Hospitals Birmingham NHS Foundation Trust, Birmingham Health Partners, Birmingham, UK

Abbreviations

ACLF, acute-on-chronic liver failure; AH, alcohol-related hepatitis; AST, aspartate aminotransferase; AVB, acute variceal bleeding; GIB, gastrointestinal bleeding; HE, hepatic encephalopathy; INR, international normalized ratio; LB, liver biopsy; LT, liver transplantation; MELD, model for end-stage liver disease; MV, multivariate analysis; NI, non-invasive; NSBBs, non-selective beta-blockers; PT, prothrombin time; sHR, subdistribution hazard ratio; sHR, subdistribution hazard ratio; TIPS, transjugular intrahepatic portosystemic shunt; UV, univariate analysis.

Conflicts of interest

MR and Christophe Bureau (CB) are speakers for Gore. Dominique Thabut (DT) is a speaker for Gore, Gilead, and Abbvie, and has a consulting role for Alfasigma. The other authors have no conflicts of interest that pertain to this work.

Please refer to the accompanying ICMJE disclosure forms for further details.

Authors' contributions

Study conception: MAR, DT. Data collection: MAR. Statistical analysis: OD, PS. Drafting the manuscript: MAR. Critical review of the manuscript: all authors.

Data availability

Data available on justified request.

Acknowledgements

The authors thank Maxime Gasperment for proofreading.

Supplementary data

Supplementary data to this article can be found online at https://doi.org/10.1016/j.jhepr.2025.101611.

References

Author names in bold designate shared co-first authorship.

[1] García-Pagán JC, Caca K, Bureau C, et al. Early TIPS (Transjugular intrahepatic portosystemic shunt) cooperative study group. Early use of TIPS in patients with cirrhosis and variceal bleeding. N Engl J Med 2010;362:2370–2379.

[2] Garcia-Pagán JC, Di Pascoli M, Caca K, et al. Use of early-TIPS for high-risk variceal bleeding: results of a post-RCT surveillance study. J Hepatol 2013;58:45–50.

[3] Nicoară-Farcău O, Han G, Rudler M, et al. Preemptive TIPS Individual Data Metanalysis, International Variceal Bleeding Study and Baveno Cooperation Study groups. Effects of early placement of transjugular portosystemic shunts in patients with high-risk acute variceal bleeding: a meta-analysis of individual patient data. Gastroenterology 2021;160:193–205.e10.

[4] Nicoară-Farcău O, Han G, Rudler M, et al. Pre-emptive TIPS Individual Data Metanalysis, International Variceal Bleeding Study and Baveno Cooperation Study groups. Pre-emptive TIPS in high-risk acute variceal bleeding. An updated and revised individual patient data meta-analysis. Hepatology 2024;79:624–635.

[5] Trebicka J, Gu W, Ibáñez-Samaniego L, et al. International variceal bleeding observational study group and Baveno cooperation. Rebleeding and mortality risk are increased by ACLF but reduced by pre-emptive TIPS. J Hepatol 2020;73:1082–1091.

[6] Hernández-Gea V, Procopet B, Giráldez Á, et al. International variceal bleeding observational study group and Baveno cooperation. Preemptive-TIPS improves outcome in high-risk variceal bleeding: an observational study. Hepatology 2019;69:282–293.

[7] Rudler M, Cluzel P, Corvec TL, et al. Early-TIPSS placement prevents rebleeding in high-risk patients with variceal bleeding, without improving survival. Aliment Pharmacol Ther 2014;40:1074–1080.

[8] Louvet A, Labreuche J, Artru F, et al. Combining data from liver disease scoring systems better predicts outcomes of patients with alcoholic hepatitis. Gastroenterology 2015;149:398–406. e8.quiz e16-7.116.

[9] Louvet A, Naveau S, Abdelnour M, et al. The Lille model: a new tool for therapeutic strategy in patients with severe alcoholic hepatitis treated with steroids. Hepatology 2007;45:1348–1354.

[10] Rudler M, Mouri S, Charlotte F, et al. Prognosis of treated severe alcoholic hepatitis in patients with gastrointestinal bleeding. J Hepatol 2015;62:816–821.

[11] Louvet A, Labreuche J, Dao T, et al. Effect of prophylactic antibiotics on mortality in severe alcohol-related hepatitis: a randomized clinical trial. JAMA 2023;329:1558–1566.

[12] Mathurin P, Louvet A, Duhamel A, et al. Prednisolone with vs without pentoxifylline and survival of patients with severe alcoholic hepatitis: a randomized clinical trial. JAMA 2013;310:1033–1041.

[13] Thursz MR, Richardson P, Allison M, et al. STOPAH Trial. Prednisolone or pentoxifylline for alcoholic hepatitis. N Engl J Med 2015;372:1619–1628.

[14] Bouzbib A, Hernández-Gea V, Bataller R, et al. International Variceal Bleeding Observational Study Group; Baveno Cooperation. Alcohol-related liver disease phenotype impacts survival after an acute variceal bleeding episode. Liver Int 2023;43:1548–1557.

[15] Crabb DW, Bataller R, Chalasani NP, et al. NIAAA Alcoholic Hepatitis Consortia. Standard definitions and common data elements for clinical trials in patients with alcoholic hepatitis: recommendation from the NIAAA Alcoholic Hepatitis Consortia. Gastroenterology 2016;150:785–790.

[16] de Franchis R. Evolving consensus in portal hypertension. Report of the Baveno IV consensus workshop on methodology of diagnosis and therapy in portal hypertension. J Hepatol 2005;43:167–176.

[17] de Franchis R, Baveno V Faculty. Revising consensus in portal hypertension: report of the Baveno V consensus workshop on methodology of diagnosis and therapy in portal hypertension. J Hepatol 2010;53:762–768.

[18] de Franchis R, VI Faculty Baveno. Expanding consensus in portal hypertension: report of the Baveno VI consensus workshop: stratifying risk and individualizing care for portal hypertension. J Hepatol 2015;63:743–752.

[19] de Franchis R, Bosch J, Garcia-Tsao G, et al. Baveno VII faculty. Baveno VII - renewing consensus in portal hypertension. J Hepatol 2022;76:959–974.

[20] Louvet A, Labreuche J, Moreno C, et al. QuickTrans trial study group. Early liver transplantation for severe alcohol-related hepatitis not responding to medical treatment: a prospective controlled study. Lancet Gastroenterol Hepatol 2022;7:416–425.

[21] Altamirano J, López-Pelayo H, Michelena J, et al. Alcohol abstinence in patients surviving an episode of alcoholic hepatitis: prediction and impact on long-term survival. Hepatology 2017;66:1842–1853.

[22] Rudler M, Hernández-Gea V, Procopet BD, et al. For International Variceal Bleeding Observational Study Group: a Baveno Cooperation. Hepatic encephalopathy is not a contraindication to pre-emptive TIPS in high-risk patients with cirrhosis with variceal bleeding. Gut 2023;72:749–758.

Keywords: Cirrhosis; Portal hypertension; Acute variceal bleeding; TIPS; Severe alcohol-related hepatitis.

Received 18 March 2025; received in revised form 22 September 2025; accepted 25 September 2025; Available online 29 September 2025

Journal of Hepatology, Volume *7*

Supplemental information

Pre-emptive TIPS should be considered in high-risk patients with both acute variceal bleeding and severe alcohol-related hepatitis

Marika Rudler, Virginia Hernández-Gea, Hélène Larrue, Charlotte Bouzbib, Bogdan Procopet, Anna Baiges, Fanny Turon, Candido Villanueva, Agustin Albillos, Edilmar Alvarado Tapias, Lise Lott Gluud, Michael Praktiknjo, Joan Genesca, Meritxell Ventura-Cots, Ares Villagrasa, Susanna Rodrigues, Sarah Mouri, Álvaro Giráldez-Gallego, Helena Masnou Ridaura, Wim Laleman, Christophe Bureau, Marie-Angèle Robic, Lukas Hartl, Luis Tellez, Alexander Zipprich, Nuria Canete, Philippe Sultanik, Olivier Deckmyn, Mattias Mandorfer, Marco Senzolo, Filippo Schepis, Dhiraj Tripathi, Juan Carlos Garcia Pagan, Dominique Thabut, and a study by the Baveno Cooperation

Pre-emptive TIPS should be considered in high-risk patients with both acute variceal bleeding and severe alcohol-related hepatitis☆

Marika Rudler, Virginia Hernandez Gea, Hélène Larrue, Charlotte Bouzbib, Bogdan Procopet, Anna Baiges, Fanny Turon, Candido Villanueva, Agustin Albillos, Edilmar Alvarado Tapias, Lise Lott Gluud, Michael Praktiknjo, Joan Genesca, Meritxell Ventura-Cots, Ares Villagrasa, Susanna Rodrigues, Sarah Mouri, Álvaro Giráldez-Gallego, Helena Masnou Ridaura, Wim Laleman, Christophe Bureau, Marie-Angèle Robic, Lukas Hartl, Luis Tellez, Alexander Zipprich, Nuria Canete, Philippe Sultanik, Olivier Deckmyn, Mattias Mandorfer, Marco Senzolo, Filippo Schepis, Dhiraj Tripathi, Juan Carlos Garcia Pagan, Dominique Thabut, a study by the Baveno Cooperation

Table of contents

Table S1……………………………………………………………………………………2

Table S2……………………………………………………………………………………3

Table S3……………………………………………………………………………………4

Table S4……………………………………………………………………………………5

Table S5……………………………………………………………………………………6

Supplementary Material 1……………………………………………………………..7

Fig. S1…………………………………………………………………………………...8

Fig. S2…………………………………………………………………………………...9

Fig. S3…………………………………………………………………………………...9

Fig. S4……………………………………………………………………………………10

Table S1: starting date for EuroTIPS database according to center.

Pitié-Salpêtrière	4/12/2020
Barcelona	10/07/2020
Birmingham	20/01/2021
Toulouse	17/12/2020
Leuven	22/07/2022
Vienna	11/12/2020
Münster	16/01/2020
Padova	16/01/2020
Modena	22/01/2021

Table S2: baseline characteristics of patients with definite or probable AH

Characteristic	Definite AH n=80	Probable AH n=62	p
**Age**	52 (44 – 57)	55 (48 – 61)	0.03
**Male Sex, %**	67 (83)	52 (84)	0.99
**AVB as first decompensating event n (%)**	32 (40)	23 (37)	0.86
**Ascites at admission n (%)**	53 (66)	41 (66)	0.99
**HE at admission n (%)**	38 (48)	30 (48)	0.99
**Shock at admission n (%)**	40 (50)	21 (36)	0.18
**Infection at admission n (%)**	17 (23)	18 (36)	0.18
**Hemoglobin (g/L)**	8.1 (6.7 – 9.2)	7.9 (6.4 – 9.2)	0.21
**Platelets count (G/L)**	86 (48 – 121)	74 (50 – 97)	0.01
**INR**	2.20 (1.82 – 2.62)	2.20 (1.87 – 2.54)	0.84
**Bilirubin (μmol/L)**	131 (77-249)	103 (72-145)	0.08
**Albumin g/L**	26 (23-30)	25 (22 – 26)	0.05
**Creatinine μmol/L**	66 (54 – 101)	68 (66 – 71)	0.77
**MELD score**	23.7 (21.0 – 29.1)	23.3 (21.5 – 26.8)	0.74
**Child-Pugh class B/C n (%)**	9/71 (11/89)	8/54 (13/87)	0.80

Student's t test was used for group comparisons of normally distributed continuous variables. Group comparisons of categorical variables were performed using Chi-squared test. A p value <0.05 was considered significant. Abbreviations: AH, alcohol-related hepatitis; AVB, acute variceal bleeding; HE, hepatic encephalopathy; INR, international normalized ratio; PT, prothrombin time ratio; MELD, model for end stage liver disease;

Table S3: diagnosis of AH according to centers

	Definite AH<br>n=80	Probable AH<br>n=62	p
			0.01
Pitié-Salpêtrière (n=69)	61 (88%)	8 (12%)	
Spain (n=35)	9 (25%)	26 (75%)	
Toulouse (n=15)	6 (40%)	9 (60%)	
Leuven (n=5)	3 (60%)	2 (40%)	
Cluj (n=4)	0 (0%)	4 (100%)	
Others (n=14)	1 (7%)	13 (93%)	

Abbreviations: AH, alcohol-related hepatitis

Table S4: Baseline MELD score and bilirubin of patients included in randomized controlled trials on AVB or alcohol-related hepatitis

Study	Clinical situation	MELD score	Bilirubin (μmol/L)
Garcia-Pagan, NEJM 2010	*pTIPS in AVB*	*15.5*	*63*
Lv, Lancet GE 2019	*pTIPS in AVB*	*14*	*21*
Dunne, APT 2023	*pTIPS in AVB*	*15.5*	*68*
Louvet, JAMA 2023	*Alcohol-related hepatitis*	*25*	*272*
Thursz, NEJM 2015	*Alcohol-related hepatitis*	*21*	*290*
Our study	*pTIPS & Alcohol-related hepatitis*	*24*	*130*

pTIPS, preemptive TIPS; AVB; acute variceal bleeding

Table S5: Cumulative incidence of death during follow-up in patients treated with pTIPS or Endo+drugs (LT competing event)

Characteristic	Time 42	Time 90	Time 180	p-value[1]
LT				
Group				0.3
Endo+drugs	1.1% (0.09%, 5.4%)	1.1% (0.09%, 5.4%)	2.4% (0.45%, 7.6%)	
TIPS	6.9% (1.7%, 17%)	9.3% (2.9%, 20%)	9.3% (2.9%, 20%)	
death				
Group				0.2
Endo+drugs	30% (21%, 40%)	35% (25%, 45%)	38% (28%, 48%)	
TIPS	16% (7.0%, 29%)	16% (7.0%, 29%)	24% (12%, 37%)	

[1] Gray's Test

The Fine and Gray model was used and comparison between patients treated with pTIPS or Endo+drugs was performed using Gray's test.

Abbreviations: LT, Liver transplantation; pTIPS, preemptive transjugular intrahepatic portosystemic shunt;

Supplementary Material 1: Management of AH

Overall, 69/142 patients (49%) patients were treated with corticosteroids, without any difference between the 2 groups (55% (pTIPS) and 46% (Endo+drugs) groups, respectively, p=0.30, see Table A below). The proportion of patients who were treated with corticosteroids was significantly higher patients with definite AH compared to patients with probable AH (70 vs 21%, p<0.001). There was no significant difference in terms of MELD, bilirubin level or infection at admission. The median duration of corticosteroids treatment was similar between the TIPS and Endo+drugs groups (7 (7-28 days) vs 28 (13-28) days, p=0.09 and not different in patients with definite or probable AH (28 days (7-28 days), vs 7 (6.5-32 days, p=0.58)).

Table A: Management of AH

	Whole cohort n=142	pTIPS n=47	Endo+Drugs n=95	p
Corticosteroids n (%)	69 (49)	26 (55)	43 (46)	0.30
Number of patients treated with 28 days of corticosteroids (%)	30 (21)	11 (23)	19 (20)	0.90
Development of infection after corticosteroids therapy	37 (36)	13 (31)	24 (40)	0.30
Lille score calculation n (%)*	56 (81)	20 (77)	36 (84)	0.50
Lille score	0.36 (0.17-0.67)	0.44 (0.17-0.59)	0.35 (0.18-0.67)	0.90
Lille score >0.45	24 (43)	10 (50)	14 (39)	0.40

*in patients who were treated with corticosteroid therapy

Student's t test was used for group comparisons of normally distributed continuous variables. Group comparisons of categorical variables were performed using Chi-squared test. A p value <0.05 was considered significant.

Abbreviations: AH, alcohol-related hepatitis; pTIPS, preemptive transjugular intrahepatic portosystemic shunt

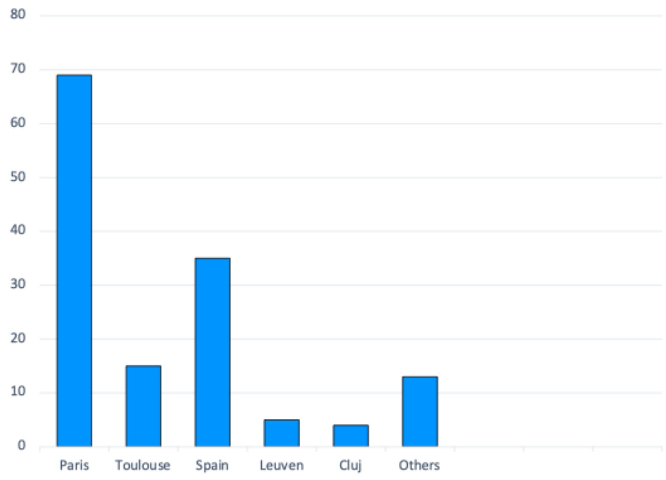

Supplementary Figure 1

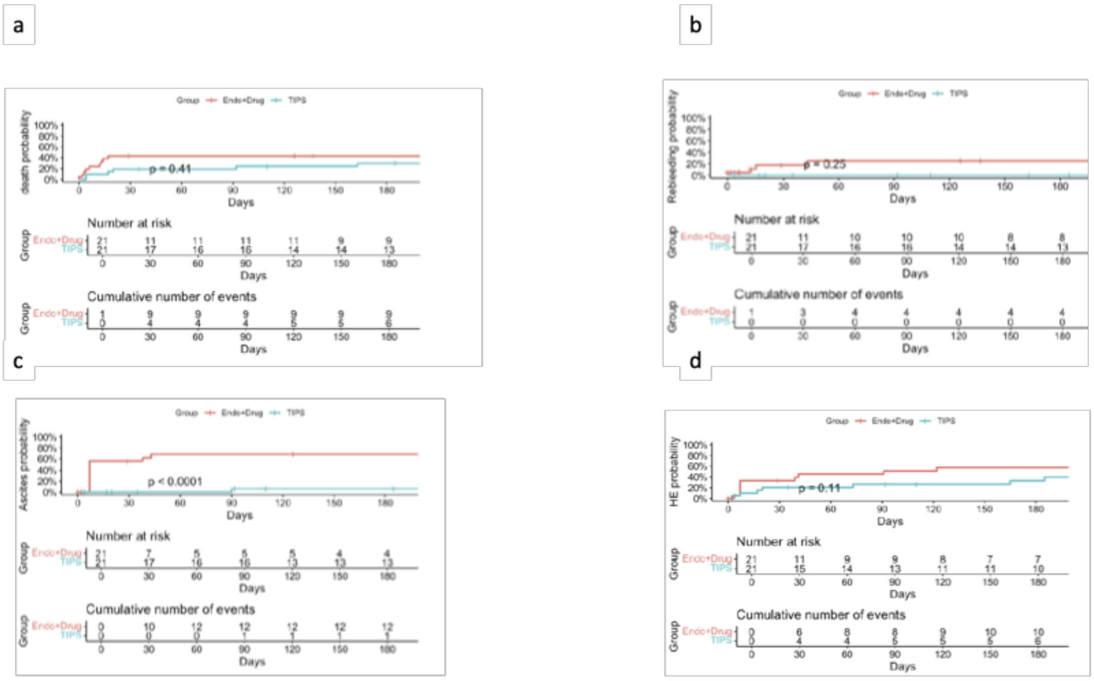

Supplementary Figure 2

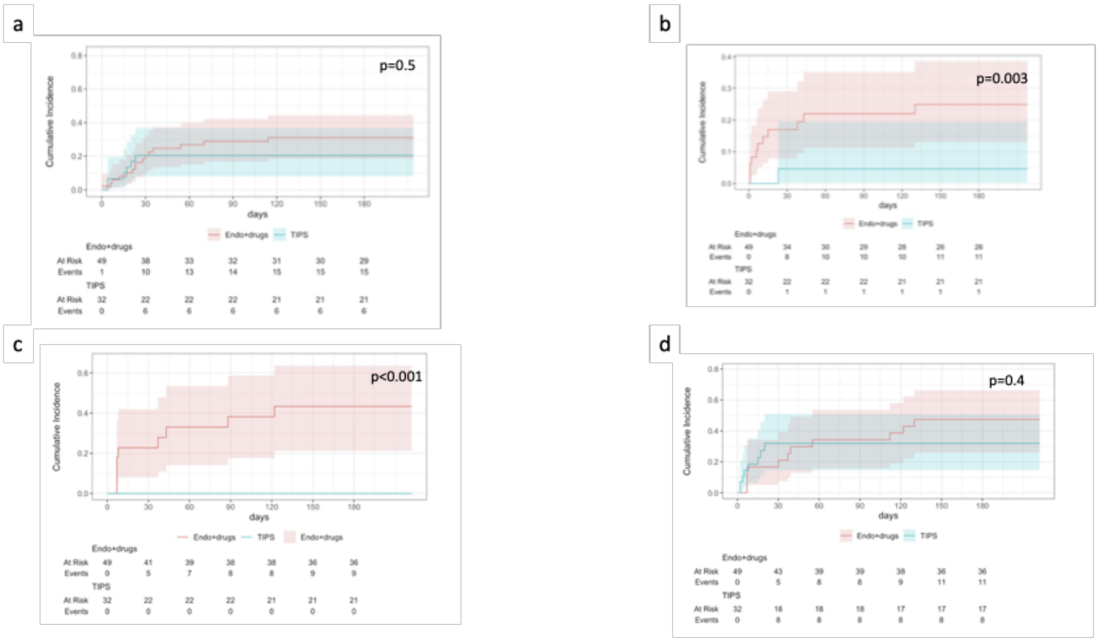

Supplementary Figure 3

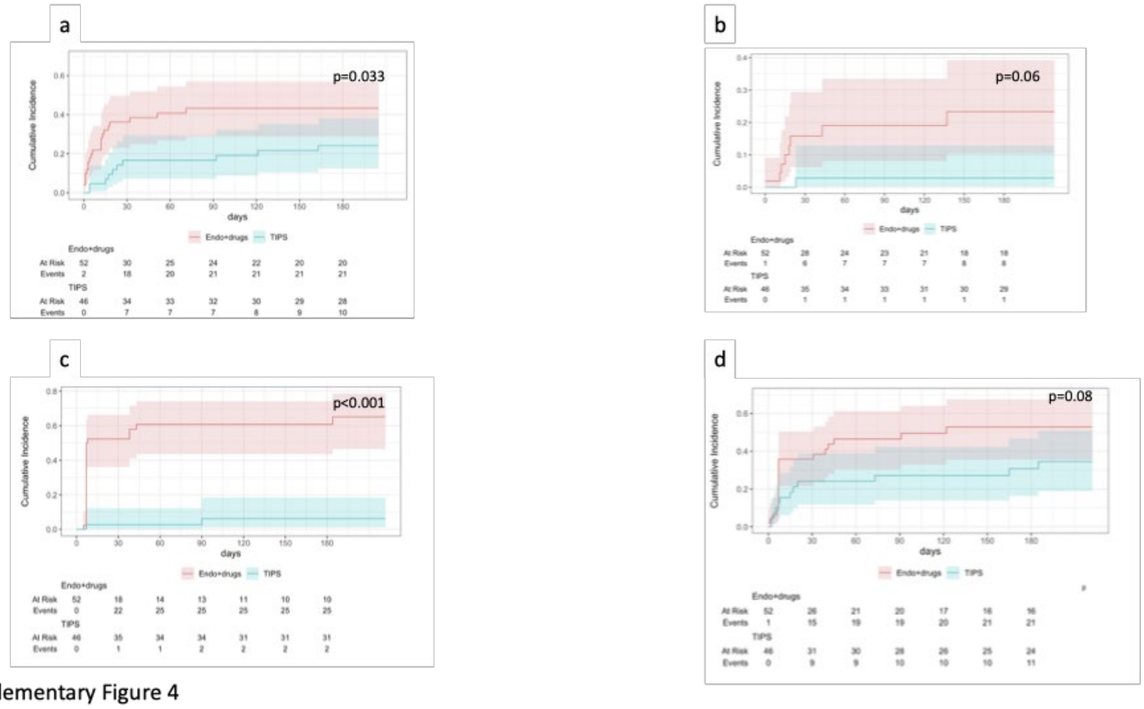

Supplementary Figure 4
